# Energy extraction, or lack thereof

**DOI:** 10.1038/s41598-023-37451-z

**Published:** 2023-07-17

**Authors:** Nishanth Gudapati

**Affiliations:** Department of Mathematics, Clark University, Worcester, 01610 MA USA

**Keywords:** Astronomy and astrophysics, Applied mathematics

## Abstract

The problem of stability of rotating black holes is the subject of a long standing research program since the 1960s and remains an unresolved problem in general relativity. A major obstacle in the black hole stability problem is that the energy of waves propagating through rotating black holes spacetimes is not necessarily positive-definite, due to the so called ergo-region. This is a serious complication that limits the efficacy of most mathematical techniques. In this expository article, we report that, despite the ergo-region, there exists a positive-definite *total energy* for axisymmetric Maxwell, gravitational and electrovacuum perturbations of Kerr and Kerr–Newman black hole spacetimes.

## Introduction

Einstein’s general theory of relativity was a great revelation of 20th century science that provided a rigorous geometric basis for ideas such as the equivalence principle and the mass. It also predicted black holes and gravitational waves. Mathematically, the Einstein equations for general relativity can be represented as

1 $$\begin{aligned} \bar{R}_{\mu \nu } =0, \quad (\bar{M}, \bar{g}), \end{aligned}$$

where $\bar{R}_{\mu \nu }$ is the Ricci curvature of the $3+1$ Lorentzian manifold $(\bar{M}, \bar{g}).$ Despite their simple appearance, in practice, these equations are a system of highly complex nonlinear partial differential equations. As a consequence, several mathematical and theoretical problems, such as the black hole stability problem, are wide open. This problem is the subject of this Letter.

Black holes are exotic objects in the universe that provide a scientific basis for exotic notions like wormholes, time travel, the big-bang etc. In a major recent development, black holes have been experimentally observed. Mathematically, black holes are solutions of the Einstein equations (1) for general relativity. In order to establish the physical relevance of black holes that occur in Einstein’s theory, it is crucial to establish that those black holes are stable under the perturbations of the Einstein equations (‘the black hole stability problem’). In particular, the Kerr family of black holes is an important two parameter (*a*, *m*) (where *a* represents the specific angular-momentum and *m* represents the mass) family of solutions of the Einstein equations that forms the genesis of the black hole stability problem.

Indeed, ever since Einstein famously published his equations for general relativity (1) in 1915 and the discovery of the Schwarzschild metric shortly thereafter, there was a general expectation that there should be a solution that represents massive, rotating black hole spacetimes. As such, attempts were made by H. Weyl, J. Ernst and A. Papapetrou to find such a solution. In 1963, when R. Kerr arrived at such a solution there was soon a wide consensus that one had arrived at the right solution. Subsequently, mathematicians and physicists conjectured that the Kerr family represents the unique asymptotically-flat solution of the stationary Einstein equations (‘the black hole uniqueness problem’) and that it is stable under perturbations with respect to the dynamical Einstein equations for general relativity (‘the black hole stability problem’). Subsequently, several stalwarts in the past have contributed to the study of these problems. These results are streamlined and summarized in the classical monograph of Chandrasekhar^1^. In recent times, the black hole stability problem is under intense investigation in the mathematics community (see e.g.,^2–5^)

**Figure 1:**
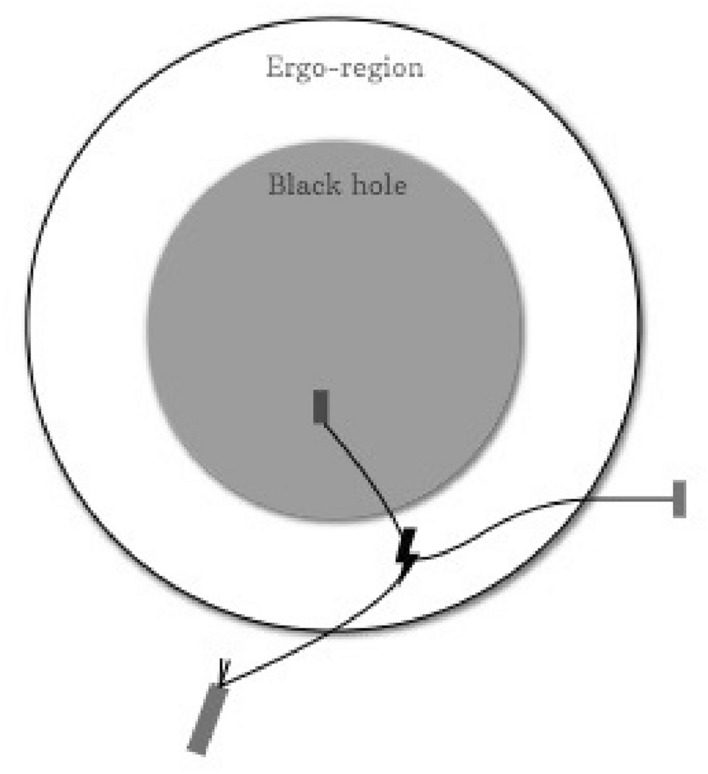
Penrose process or energy extraction for a rotating Kerr black hole.

An important first step in the resolution of the black hole stability problem is to study the Maxwell equations and the linear perturbative theory of the Einstein equations around the Kerr black hole spacetimes. However, a peculiar property of Kerr black holes $( a \ne 0 )$ is that one is always surrounded by a so called ergo-region. In the ergo-region, an object cannot remain stationary and is forced to move along with the rotating black hole. A surprising feature of the ergo-region can be explained using Roger Penrose’s gedanken experiment (see Fig. 1). Suppose we throw an object into a Kerr black hole in such a way that it splits into two pieces (as shown in Fig. 1), where one piece enters the black hole and the other exits the ergo-region. The piece that exits can have higher energy than the original object. This is not a violation of the conservation of energy because the piece that goes into the black hole carries ‘negative’ energy.

This phenomenon can also be seen for linear scalar waves propagating on Kerr black holes^6,7^ (see also^8^). In other words, linear scalar waves propagating on a Kerr black hole, do not necessarily have a positive-definite energy. By analogy with Penrose’s gedanken experiment, this results in a phenomenon where it appears as though the black hole is emitting energy. This phenomenon is referred to as the Penrose process or superradiance or energy extraction.

This feature of the ergo-region has important implications for the mathematical problem of the stability of Kerr black hole spacetimes. The aforementioned model of waves propagating on a Kerr black hole is analogous to the Maxwell and linear perturbations of the Einstein equations around a Kerr black hole spacetime. As it can be intuitively imagined, the stability of a system is closely linked to the decay of perturbations, which in turn is associated to ‘loss’. The fact that a Kerr black hole effectively ‘emits’ energy, counteracts the ‘decaying’ nature of the perturbations. In fact, in axial symmetry, it can be established that, if the energy is not positive, it will blow up exponentially in time, implying instability of Kerr black hole spacetimes^9^. Furthermore, the methods of partial differential equations that establish decay are typically based on a positive energy. Likewise, in the Lyapunov theory of stability of dynamical systems, a notion of energy and its positive-definiteness serve as important criteria for various notions of stability. Thus, the lack of a positive-definite and conserved energy is a significant issue that limits the strength of various mathematical methods in the resolution of the black hole stability problem.

In this Letter, we report that, despite the ergo-region, there exists a positive-definite *total energy* for axisymmetric perturbations of Kerr and Kerr–Newman black hole spacetimes.

## Maxwell equations in Kerr black hole spacetimes

In the case of axisymmetric linear scalar waves propagating in a Kerr black hole spacetime, the problem of the ergo-region disappears on account of the fact that Kerr black holes are also axially symmetric. To see this, consider the linear wave equation propagating in the Kerr black hole spacetime $(\overline{M}{}, \bar{g})$

2 $$\begin{aligned} \square _{\bar{g}} u = 0, \quad (\overline{M}, \bar{g}), \end{aligned}$$

where $\square _{\bar{g} } \,:\,= \frac{1}{\sqrt{-\bar{g}}} \partial _\mu ( \bar{g}^{\mu \nu } \partial _\nu u)$. Let us represent the line element of the Kerr metric $(\overline{M}{}, \bar{g})$ in the ADM form:

3 $$\begin{aligned} ds^2=&\bar{g}_{ \mu \nu } dx^\mu \otimes dx^\nu \nonumber \\ =&\bar{N} dt^2 + \bar{q}_{ij} (dx^i + \bar{N}^i dt) \otimes (dx^i + \bar{N}^j dt), \quad i, j= 1, 2, 3, \end{aligned}$$

where $\bar{N}$ and $\bar{N}^i$ are the lapse and shift respectively; and $\bar{q}$ is the metric of the spacelike Riemannian manifold $\overline{\Sigma }{}$, such that $\overline{M}{} = \mathbb {R} \times \overline{\Sigma }{}.$ A simple calculation shows that the Kerr metric in Boyer–Lindquist coordinates $\{t, r, \theta , \phi \}$ can be represented in the ADM form (3). It may also be noted that $\bar{N}^\phi \ne 0$ and all other components of the shift vector $\bar{N}$ vanish. After the Legendre transformation, the Hamiltonian energy of the linear wave equation is given by

4 $$\begin{aligned} H^{\text {LW}} = \int _{\overline{\Sigma }{}} \left( \frac{1}{2}\bar{N} \bar{\mu }^{-1}_{\bar{q}} v^2 + \frac{1}{2}N \bar{\mu }_{\bar{q}} \bar{q}^{ij} \partial _i u \partial _j u + v N^i \partial _i u \right) d^3 x, \end{aligned}$$

where *v* is the conjugate momentum and $\bar{\mu }_{\bar{q}} = \sqrt{\bar{q}}.$ It may be noted that the third term in the Hamiltonian energy (4) has an indefinite sign. However, as we already remarked, since $N^\phi$, is the only non-vanishing component of the shift vector and our assumption that *u* is axially symmetric $(\partial _ \phi u \equiv 0),$ this term fortuitously drops out i.e., $v N^i \partial _i u \equiv 0.$ As a result $H^{\text {LW}}$ is positive-definite.

However, the problem of ergo-region reappears for both axisymmetric Maxwell and linearized Einstein fields propagating on Kerr black holes. In fact, one can arrange for the axisymmetric Maxwell equations that their energy density is *locally negative*. The question of whether the axisymmetric Maxwell and Einstein equations admit a positive-definite total energy (for $\vert a \vert <M$), somewhat analogous to axisymmetric linear waves, has been one of those perplexing ‘borderline’ open problems for decades.

In a recent work, we were able to resolve this long standing open problem. Specifically, we were able to establish that one can construct a positive-definite *total* energy for axisymmetric Maxwell fields propagating through sub-extremal Kerr black hole spacetimes ($\vert a \vert <M$)^10^. An interesting aspect of our result is that we are able to construct a positive-definite *total integrated* energy, even though the *local* energy density could be *negative*. Our proof relies on a Hamiltonian framework of the dimensionally reduced axisymmetric Einstein equations and certain transformations that were originally developed for the black hole uniqueness theorems by B. Carter and D.C. Robinson^11,12^.

In the following, we shall sketch our proof for the construction of a positive-definite and conserved energy for axisymmetric electromagnetic (Maxwell) fields propagating on Kerr black hole spacetimes.

Consider the Lagrangian variational principle of the Maxwell equations:

5 $$\begin{aligned} S^{\text {Max}} \,:\,= - \frac{1}{4} \int \Vert F \Vert _{\bar{g}} ^2\, \bar{\mu }_{\bar{g}}, \quad \hbox { on the Kerr metric}\ (\bar{M}, \bar{g}), \end{aligned}$$

where *F* is the Maxwell tensor derivable from the vector potential *A* and $\bar{\mu }_{\bar{g}}$ is the volume form of $(\bar{M}, \bar{g})$. It is well known that^13^, if we perform a Legendre transform of the functional $S_{\text {Max}},$ we get a Hamiltonian variational principle for the Maxwell fields:

6 $$\begin{aligned} I^{\text {Max}} \,:\,= \int \left( A_i \partial _t {\fancyscript{E}^i} - \bar{N} \fancyscript{H}^{\text {Max}} - \bar{N}^i \fancyscript{H}_i^{\text {Max}} -A_t \partial _i \fancyscript{E}^i \right) d^4 x \end{aligned}$$

over the phase space $\{ (A_i, \fancyscript{E}^i), i = 1, 2, 3 \},$ where $\{ A_i, i=1, 2, 3 \}$ are the canonical ‘position’ variables and $\{ \fancyscript{E}^i, i = 1, 2, 3 \}$ are the canonical ‘momentum’ variables (‘conjugate momenta’); $\fancyscript{H}^{\text {Max}}$ and $\fancyscript{H}_i^{\text {Max}}$ are defined as

7 $$\begin{aligned} \fancyscript{H}^{\text {Max}} \,:\,=&\frac{1}{2}\bar{q}^{ij} \bar{\mu }^{-1}_{\bar{q}} \left( \fancyscript{E}^i \fancyscript{E}^j + \fancyscript{B}^i \fancyscript{B}^j \right) \nonumber \\ \fancyscript{H}_i^{\text {Max}} \,:\,=&- \varepsilon _{ijk} \fancyscript{E}^j \fancyscript{B}^k, \end{aligned}$$

where $\fancyscript{E}^i$ and $\fancyscript{B}^i = \frac{1}{2}\varepsilon ^{ijk} (\partial _j A_k - \partial _k A_j)$ are the ‘electric’ and ‘magnetic’ components of *F* respectively.

Let us define the Hamiltonian energy functional $H^{\text {Max}}$ over the phase space $X^{\text {Max}} \,:\,= \{ A_i, \fancyscript{E}^i \}$ as follows

8 $$\begin{aligned} H^{\text {Max}} \,:\,= \int _{\overline{\Sigma }{}} \left( \frac{1}{2}\bar{N} \bar{q}_{ij} \bar{\mu }^{-1}_{\bar{q}} (\fancyscript{E}^i \fancyscript{E}^j + \fancyscript{B}^i \fancyscript{B}^j) - N^i \varepsilon _{ijk} \fancyscript{E}^j \fancyscript{B}^k \right) d^3x. \end{aligned}$$

In (8), It may be seen readily that it has an indefinite sign, even in axial symmetry. Recall the Maxwell constraints (i.e., the Gauss Law):

9 $$\begin{aligned} \partial _i \fancyscript{E}^i =0, \quad \partial _i \fancyscript{B}^i =0. \end{aligned}$$

In view of the fact that the quotient space $\Sigma \,:\,= \overline{\Sigma }{}/SO(2)$ is simply connected, we can now introduce the variables $\lambda$ and $\eta$ (‘the twist potentials’) such that

10 $$\begin{aligned} \fancyscript{E}^a = \varepsilon ^{ab} \partial _b \eta \quad \text {and} \quad \fancyscript{B}^a = \varepsilon ^{ab} \partial _b \lambda , \end{aligned}$$

taking advantage of the Maxwell constraint equations (9) on the quotient space $\Sigma = \overline{\Sigma }{}/SO(2)$ and form the new phase space *X* : 

11 $$\begin{aligned} X \,:\,= \big \{ ( \lambda , v), (\eta , u) \big \}, \quad \hbox {where} v \,:\,= - \fancyscript{E}^\phi \hbox {and} u \,:\,= \fancyscript{B}^\phi . \end{aligned}$$

The variables *v* and *u* are the conjugate momenta of $\lambda$ and $\eta$ respectively so that $(\lambda , v)$ and $(\eta , u)$ are canonical pairs. In these new variables, the energy functional $H^{\text {Max}}$ can now be reduced to

12 $$\begin{aligned} H^{\text {Alt}} \,:\,=&\int _{\Sigma } e^{\text {Alt}} d^2x, \quad \text {where} e^{\text {Alt}} \text { is defined as,} \end{aligned}$$

13 $$\begin{aligned} e^{\text {Alt}} \,:\,=&\frac{1}{2}N e^{2 \gamma } \bar{\mu }^{-1}_q (v^2 + u^2) + \frac{1}{2}N \bar{\mu }_q q^{ab} e^{-2\gamma } (\partial _a \eta \partial _b \eta + \partial _a \lambda \partial _b \lambda ) + N e^{-4\gamma } \bar{\mu }_q q^{ab} \partial _a \omega \partial _b \eta \lambda , \end{aligned}$$

upto a few boundary terms, which vanish under suitable boundary conditions (for example, when the Maxwell fields are compactly supported). In this reduction, we used a combination of the ADM metric and the Weyl–Lewis–Papapetrou form of the background Kerr metric:

14 $$\begin{aligned} \bar{g} = e^{-2\gamma } \left( -N^2 dt^2 + q_{ab} (dx^a + N^a dt) (dx^b + N^b dt) \right) + e^{2 \gamma } \left( d \phi + \fancyscript{A}_0 dt + \fancyscript{A}_a dx^a \right) . \end{aligned}$$

In these coordinates, the norm of the axial killing vector field of the Kerr metric, $\Vert \partial _\phi \Vert =\,:\,e^{2 \gamma }.$ Again notice that $H^{\text {Alt}}$ has an indefinite sign. In fact, we can arrange that the energy density $e^{\text {Alt}}$ is locally negative inside the ergo-region (see Sect. 2 in Ref.^10^).

While the positivity problem is not immediately solved in this new phase space *X*, the advantage of this formulation is that it allows transformation that results in a positive-definite, regularized, Hamiltonian. This is facilitated by the Carter–Robinson identities^11,12^. Carter–Robinson identities were originally developed in the context of the black hole uniqueness problem. The essence of the black hole uniqueness problem is that, under some regularity and asymptotic conditions, the Kerr black hole spacetime is the unique stationary vacuum black hole spacetime. Carter–Robinson identities were part of the early efforts towards the resolution of this problem. These methods focused on obtaining the uniqueness of Kerr black hole spacetimes, in the case of axial symmetry, using the theory of elliptic partial differential equations, where positive-definite functionals play an important role.

The following is the Carter–Robinson identity for Maxwell fields. We refer the reader to Sect. 3.3 in^10^ for the Einstein–Maxwell fields, which is considerably more elaborate.

15 $$\begin{aligned}&N \bar{\mu }_q q^{ab} e^{-2 \gamma } (\partial _a \eta \partial _b \eta + \partial _a \lambda \partial _b \lambda ) + 2 N \bar{\mu }_q q ^{ab} e^{-4 \gamma } \partial _a \omega \partial _b \eta \lambda + L_1 ( \frac{1}{2}\eta ^2 + \frac{1}{2}\lambda ^2) - \lambda \eta L_2 \nonumber \\&\qquad + \frac{1}{2}\partial _b (-2N \bar{\mu }_q q^{ab} e^{-4 \gamma } \partial _a \omega \eta \lambda + N \bar{\mu }_q q^{ab} \partial _a (e^{-2 \gamma }) (\lambda ^2 + \eta ^2 )) \nonumber \\&\quad = \frac{1}{2}N e^{2 \gamma } \bar{\mu }_q q^{ab} \left( \partial _a ( e^{-2\gamma } \lambda ) \partial _b (e^{-2\gamma } \lambda ) + \partial _a (e^{-2\gamma } \eta ) \partial _b (e^{-2\gamma } \lambda ))\right) \nonumber \\&\qquad + \frac{1}{2}N e^{-2\gamma } \bar{\mu }_q q^{ab} \left( (\partial _a \eta + \lambda e^{-2\gamma } \partial _a \omega ) ( \partial _b \eta + \lambda e^{-2\gamma } \partial _b \omega ) + (\partial _a \lambda - \eta e^{-2\gamma } \partial _a \omega ) (\partial _b \lambda - \eta e^{-2\gamma } \partial _a \omega ) \right) , \end{aligned}$$

where the quantities $\gamma$ and $\omega$ are the quantities associated to the background Kerr black hole spacetimes and satisfy the following equations,

16 $$\begin{aligned} L_1 \,:\,= \,\,&\frac{1}{2}e^{-2\gamma } \left( 4 \partial _b (N \bar{\mu }_q q^{ab} \partial _a \gamma ) + 2 N e^{-4\gamma } \bar{\mu }_q q^{ab} \partial _a \omega \partial _b \omega \right) =0, \end{aligned}$$

17 $$\begin{aligned} L_2 \,:\,=&- \partial _b \left( N \bar{\mu }_q q^{ab} e^{-4\gamma } \partial _a \omega \right) =0. \end{aligned}$$

Let us make a few remarks on the identity (15). Firstly notice that, the first line in the left hand side of the equation is the same as the potential energy in (12), which has an indefinite sign. However, crucially, this can be transformed into a positive-definite quantity on the right hand side, up to a boundary term. The boundary term vanishes for suitable boundary conditions, for example when the Maxwell fields are compactly supported. A similar argument was used by Carter–Robinson in their black hole uniqueness theorems, but we have now used this identity for the dynamical black hole stabilty problem.

Therefore, we have a positive-definite energy comprising of the right hand side of (15). For convenience, let us define the new phase space

$$\begin{aligned} \underline{X}{}\,:\,=\big \{(\underline{\lambda }{}, \underline{v}{} ), ( \underline{\eta }{}, \underline{u}{}) \big \}, \end{aligned}$$

where

$$\begin{aligned} \underline{\eta }{} \,:\,= e^{-\gamma } \eta , \quad \underline{u}{} \,:\,= e^\gamma u, \quad \underline{\lambda }{} \,:\,= e^{-\gamma } \lambda , \quad \underline{v}{} \,:\,= e^\gamma v. \end{aligned}$$

The positive-definite energy can now be represented as $H^{\text {Reg}} = \int _{\Sigma } e^{\text {Reg}} d^2x$, where $e^{\text {Reg}}$ is

18 $$\begin{aligned} e^{\text {Reg}} \,:\,=&\frac{1}{2}N \bar{\mu }^{-1}_q (\underline{u}{}^2 + \underline{v}{}^2) + \frac{1}{4} N \bar{\mu }_q q^{ab} \left( 2 \partial _a \gamma \partial _b \gamma + \frac{1}{2}e^{-4\gamma } \partial _a \omega \partial _b \omega \right) ( \underline{\lambda }{}^2 + \underline{\eta }{}^2) \nonumber \\&\frac{1}{2}N \bar{\mu }_q q^{ab} \Big ( (\partial _a \underline{\lambda }{} - \frac{1}{2}\underline{\eta }{} e^{-2\gamma } \partial _a \omega )(\partial _b \underline{\lambda }{} - \frac{1}{2}\underline{\eta }{} e^{-2\gamma } \partial _b \omega ) + (\partial _a \underline{\eta }{} + \frac{1}{2}\underline{\lambda }{} e^{-2\gamma } \partial _a \omega )(\partial _b \underline{\eta }{} + \frac{1}{2}\underline{\lambda }{} e^{-2\gamma } \partial _b \omega ) \Big ). \end{aligned}$$

Importantly, these transformations do not destroy Hamiltonian nature of the energy i.e., the positive-definite quantity $H^{\text {Reg}}$ is also a Hamiltonian for the phase space $\underline{X}{}$:

19 $$\begin{aligned} \partial _t \underline{\eta }{} = \frac{\delta H^{\text {Reg}}}{\delta \underline{u}{}}, \quad \partial _t \underline{\lambda }{} = \frac{\delta H^{\text {Reg}}}{\delta \underline{v}{}}, \quad \partial _t \underline{u}{} = - \frac{\delta H^{\text {Reg}}}{\delta \underline{\eta }{}}, \quad \partial _t \underline{v}{} = - \frac{\delta H^{\text {Reg}}}{\delta \underline{\lambda }{}}. \end{aligned}$$

Our construction can be schematically represented as follows:

20 $$\begin{aligned} (X^{\text {Max}}, H^{\text {Max}}) \rightarrow (X, H^{\text {Alt}}) \rightarrow (\underline{X}{}, H^{\text {Reg}}), \end{aligned}$$

where $H^{\text {Reg}} \ge 0.$ Furthermore, we can construct a divergence-free vector field density *J* such that $J^t = e^{\text {Reg}}$ i.e.,

21 $$\begin{aligned} \partial _t J^t + \partial _a J^a =0, \quad a= 1, 2, \end{aligned}$$

where

22a $$J^t = H^{\text {Reg} }$$

22b $$\begin{aligned} J^a=&-N^2 q^{ab} \left( u \left( \partial _a \eta + \frac{1}{2}\lambda e^{-2\gamma } \partial _a \omega \right) + v \left( \partial _a \lambda - \frac{1}{2}\eta e^{-2 \gamma } \partial _a \omega \right) \right) . \end{aligned}$$

Furthermore, crucially, the flux of *J* through the event-horizon $\fancyscript{H}$ (which is a null hypersurface) also has a positive-definite sign i.e.,

23 $$\begin{aligned} \text {Flux} (J, \fancyscript{H}) \ge 0, \end{aligned}$$

which implies that the integrated energy flux of axisymmetric Maxwell fields is *ingoing*, into the event-horizon.

Following our results, a positive energy functional for Maxwell fields was also constructed by Prabhu–Wald^14^ using their notion of ‘canonical energy’. It turns out that the energy (18) can also be used to construct a positive energy for Newman-Penrose-Maxwell scalars on Kerr black holes^15^. Furthermore, the methods presented above are versatile and they can be extended to Maxwell fields on rapidly rotating $(\vert a \vert <M)$ Kerr–de Sitter black hole spacetimes^16^. We would like to point out that, a positive-definite energy was first constructed by Dain–de Austria^17^ for axisymmetric linearized Einstein perturbations of extremal Kerr black holes $(\vert a \vert =M)$, using the Brill mass formula^18,19^. Let us make a few remarks on the Brill mass formula. The concept of positive-definite mass-energy was a difficult problem in general relativity and remained unresolved for several decades after Einstein published his general theory of relativity in 1915. The Brill mass formula was the first demonstration that there exists a positive mass for the Einstein equations. Brill obtained a mass formula for axi-symmetric and time-symmetric Cauchy hypersurfaces by integrating the Hamiltonian constraint and noting that the boundary term provides a mass and the bulk terms are positive-definite. Subsequently, Arnowitt–Deser–Misner (ADM) obtained a more geometric notion of mass-energy (the ‘ADM’ mass^20^) and the positivity of it was established by Schoen–Yau^21,22^ and Witten^23^ in celebrated works.

The positive energy (18) presented here is a special case of our elaborate work^10^ on axisymmetric linearized Einstein–Maxwell perturbations of Kerr–Newman black hole spacetimes (it may be recalled that the first-order linearization of Einstein–Maxwell fields about the Kerr black hole background yields decoupled, purely Maxwellian fields on the Kerr background). Results analogous to the ones presented here hold for linearized Einstein perturbations of Kerr and Kerr–Newman black hole spacetimes^10,24,25^, but the mathematical methods involved are much more delicate (see “Linearized Einstein and electrovacuum perturbations”). For axisymmetric linearized Einstein perturbations, we use the linearization stability methods developed by Fischer–Marsden–Moncrief^26–28^. In particular, the construction of the energy is based on the kernel of the adjoint of the dimensionally reduced constraints. These methods, together with the Carter–Robinson identities, provide an explanation for why the transformations (20) almost magically provide a positive-definite energy, ‘out of the blue’.

## Linearized Einstein and electrovacuum perturbations

There are few fundamental differences between the gravitational perturbations and the electromagnetism on Kerr black holes. In the gravitational problem, the difficulty in the construction of a positive energy is not only due to the ergo-region—there are geometric and gauge related obstacles in addition. Consider for example the case of Schwarzschild black hole spacetimes, which do not contain the ergo-region or exhibit energy extraction phenomenon. Nevertheless, the construction of a positive-definite energy for gravitational perturbations of Schwarzschild black hole spacetimes is not trivial by any means (see e.g.,^29–31^).

In spite of the issues mentioned above, we report that there exists a positive-definite, gauge-invariant and strictly conserved total energy functional for linearized Einstein and electrovacuum perturbations of Kerr and Kerr–Newman black holes for the full sub-extremal range $\vert a, Q\vert <M$. This outcome is made possible by the special structure offered by wave maps in axial symmetry.

The Einstein equations for general relativity in axial symmetry, when represented in the Weyl–Lewis–Papapetrou gauge:

24 $$\begin{aligned} \bar{g} = e^{-2 \gamma } g + e^{2 \gamma } (d \phi + A_\nu dx^\nu )^2, \nu = 0, 1, 2, \end{aligned}$$

can be reduced to the $2+1$ dimensional Einstein wave map system, where the wave map

$$\begin{aligned} U \,:\,(M, g) \rightarrow (\mathbb {H}^2, h) \end{aligned}$$

has the negatively curved target, the hyperbolic 2-plane $\mathbb {H}^2$. Likewise, the Einstein–Maxwell equations can be reduced to the $2+1$ Einstein-wave map system, with the complex hyperbolic plane $\mathbb {H}_{\mathbb {C}}^2$ as the target manifold for the wave map.

We construct an energy using the quantity

25 $$\begin{aligned} H^{\text {Alt}} \,:\,= \int _{\Sigma } (N\, D^2 \cdot H ) \, d^2 x, \end{aligned}$$

where *H* is the dimensionally reduced Hamiltonian constraint on $\Sigma ,$ *D* is the variational derivative and *N* is the dimensionally reduced lapse in the orbit space $M \,:\,= \bar{M}/SO(2),$ *g* is the metric of *M* as in (24), $d^2x$ is the coordinate volume form in $\Sigma$. Importantly, that the quantity (25) can serve as a candidate for the energy follows from the theory of Linearization Stability, developed by Fischer–Marsden–Moncrief^26–28^.

Let us briefly discuss about the Linearization stability methods. Linearization stability concerns the smoothness of the space of the solutions of the Einstein equations for general relativity. A spacetime manifold is linearization stable within the space solutions if a linearized perturbation can in principle be extended to higher-order perturbations. In other words, consider an exact solution of the Einstein equations, this exact solution is linearization stable if all solutions of the linearized equations are tangent to the curves of exact solutions passing through the given one. In a seminal work, Fischer–Marsden^26^ established the criterion that, if the kernel of the adjoint of the linearized Einstein constraint map is trivial, then the spacetime is linearization stable. In a beautiful work, Moncrief^28^ had established a relation between Killing vector fields, the dimension of kernel of the Einstein constrain map and linearization stability, for spacetimes with a compact Cauchy hypersurface.

We borrow this insightful relation between the Killing vector field and the Kernel of the adjoint of the lineared constraint map for black hole stability problem. It is precisely the kernel of the adjoint of dimensionally reduced constraint map that allows us to construct a natural notion of energy for linearized axisymmetric perturbations of the Kerr–Newman black hole spacetime. In particular, the energy functional (25) is derived from the insight that (*N*, 0) is the kernel of the adjoint of the dimensionally reduced linearized constraints.

The quantity $H^{\text {Alt}}$ in (25) can further be transformed using the generalized Carter-Robinson identities [analogous to (20)] into a positive-definite energy functional $H^{\text {Reg}}$^10,24^. We refer the reader to Sect. 3.3 in Ref.^10^ for the full presentation of the Carter–Robinson identities. It is worth pointing out that, in the linearized Einstein problem, it becomes transparent that the underlying cause for the validity of the Carter–Robinson identities is the negative curvature of the target manifold of the wave map, the hyperbolic 2-plane $\mathbb {H}^2$^24^.

A positive-definite energy defined on a space-like Cauchy hypersurface is of limited significance for the stability problem if it is not strictly conserved in time or if the constituting fields are not regular functions. These two issues are coupled in the sense that the obstacles for strict conservation of energy are caused by the boundary terms that occur at the boundaries of the orbit space—the axes, the spatial infinity and the intersection of axes and the horizon; and it is precisely at these boundaries that the regularity issues arise. Furthermore, in contrast with the electromagnetic problem (“Maxwell equations in Kerr black hole spacetimes”), the regularity and the strict conservation of energy in the linearized Einstein (i.e., gravitational) and the electrovacuum problems are further complicated by gauge related issues.

In order to overcome these issues, we formulate the initial value problem in the harmonic gauge (Lorenz-harmonic gauge for electrovacuum perturbations of Kerr–Newman black holes), where we can establish causality and regularity of the future development using standard energy methods, and transform the perturbed fields into the Weyl–Papapetrou gauge. Firstly, we establish that the gauge-transformation as well as the gauge-transformed fields in the Weyl–Papapetrou gauge are dynamically regular at all the boundaries. Subsequently, we prove strict conservation of the positive-definite energy $H^{\text {Reg}}$ by establishing that all the boundary flux terms vanish dynamically in time^10,25^. In this context, we take advantage of the fact that the transverse-traceless tensors vanish for our geometry and thus the elliptic operators can be reduced to Poisson operators, which in turn provide the needed regularity and decay rates at the axes and infinity^10,25^.

## Summary and concluding remarks

The problem of stability of Kerr black holes is a major open problem in theoretical and mathematical studies in general relativity. Arguably, the most important obstacle in establishing the stability of Kerr black holes is the ergo-region and its effects like superradiance and energy-extraction. The effects of the ergo-region are even stronger for rapidly rotating sub-extremal Kerr black holes $(\vert a \vert <M)$. Due to this reason, most of the current and past works on the black hole stability problem are dedicated to the study of stability of Kerr black holes for small angular-momentum $(\vert a \vert \ll M)$.

Theoretically, due to the energy extraction phenomenon, the energy of perturbations can blow up in time, implying instability of Kerr black holes. In this Letter, we presented a result wherein we constructed a positive-definite energy for axisymmetric Maxwell fields propagating through Kerr black holes. Analogous results hold for axisymmetric gravitational and electrovacuum perturbations of Kerr and Kerr–Newman black hole spacetimes respectively, using much more delicate mathematical methods. These results imply a form of stability of Kerr and Kerr–Newman black hole spacetimes in axial symmetry. Since our results hold for the entire sub-extremal range of Kerr and Kerr–Newman black holes (it may be recalled that rapidly rotating, but sub-extremal, black holes are physically important), these results are expected to be a major step forward towards the resolution of the black hole stability problem. Furthermore, the methods are suitable for the study of stability of Kerr–Newman–de Sitter black holes and higher-dimensional black hole spacetimes under a suitable symmetry class.

## Data Availability

Data sharing is not applicable to this article as no datasets were generated or analysed during the current study.
